# Determinants of DMPA-SC self-care/self-injectable contraceptive uptake among modern contraceptive users in Burkina Faso: findings from the 2021 demographic and health survey

**DOI:** 10.3389/fgwh.2024.1385446

**Published:** 2024-09-05

**Authors:** Aristide Romaric Bado

**Affiliations:** Département Biomedical et Santé Publique, Institut de Recherche en Sciences de la Santé (IRSS), Centre National de la Recherche Scientifique et Technologique (CNRST), Ouagadougou, Burkina Faso

**Keywords:** determinants, DMPA-SC, contraception, DHS, Burkina Faso

## Abstract

**Introduction:**

This study aimed to identify the determinants that influence the use of DMPA-SC/Sayana Press among women who use modern contraceptive methods in Burkina Faso.

**Methods:**

This study used secondary data obtained from the 2021 Burkina Faso Demographic and Health Survey (EDSBF). The dependent variable is the use of DMPA-SC among women aged 15–49 who employ modern contraceptive methods. The descriptive analysis used percentages to describe the study variables. The Pearson chi-square test was used to assess the associations between the explanatory variables and the study variable of interest. Bivariate logistic regression was used to examine the crude odds ratios of each explanatory variable with respect to the dependent variable. The multivariate model was used to determine the net effect of each independent variable on the dependent variable. The significance levels were defined at *p* < 0.05, with corresponding confidence intervals.

**Results:**

The study revealed significant differences in the use of DMPA-SC according to age, marital status, region of residence, level of education, number of children, and involvement in contraceptive decision-making within the couple. Younger women (aged 15–29 aOR = 2.12, *p* < 0.001)) and women aged 30–39 (aOR = 1.51, *p* = 0.02) are also more likely to use DMPA-SC compared to those aged 40–49. Married women or those living with a partner [aOR = 1.93 (1.22, 3.05)] are more likely to use DMPA-SC. Women with 1–3 children are twice as likely to use DMPA-SC as those without children (aOR = 1.97, *p* = 0.02). Region and Wealth Index were significantly associated with DMPA-SC use. The Boucle du Mouhoun region showed a significantly higher likelihood of DMPA-SC use (aOR = 8.10) and women in the highest wealth group are significantly less likely to use DMPA-SC (aOR = 0.59, *p* = 0.001).

**Conclusion:**

These results demonstrated the importance of adapting interventions to account for socio-demographic, regional, and cultural differences. This will enable the provision of services to the entire female population in a fair and equitable manner, while also addressing the limitations and enhancing the understanding of the underlying factors influencing the use of DMPA-SC.

## 1 Introduction

Adopting a modern contraceptive method reduces maternal morbidity and mortality rates (1, 2). Pregnancies that occur too early, too late, too frequently, and too closely spaced are primary causes of direct obstetric complications, which account for over 70% of maternal deaths in low-income countries (3). Consequently, family planning is crucial for reducing fertility rates and maternal mortality by avoiding high-risk pregnancies and decreasing the need for abortions, thereby helping to increase women's life expectancy (4). Central to achieving the 2030 Sustainable Development Goals (SDGs), family planning has major implications in areas such as gender, employment, poverty reduction, and health (5).

Despite these benefits, contraceptives remain underused in many developing regions (6, 7). Typically, women from favourable backgrounds and living conditions exhibit higher contraceptive use, whereas women from impoverished households, who are in greater need of contraceptives, often have limited access to them (4).

The global sexual and reproductive health and rights (SRHR) agenda now prioritizes the right of women to freely choose the number and timing of their children (8). A number of recent initiatives call for the gaps in access to modern contraception to be filled, particularly when women wish to avoid pregnancy but do not have the means or use traditional methods (9). One of the flagship initiatives in this area is Family Planning 2020, a global partnership launched in 2012. It aims to add 120 million new modern contraceptive users in the world's 69 least-developed countries by 2020 (9).

For family planning to be effective, it is essential to ensure continuity in the use of contraception. However, many factors, such as cost, convenience, satisfaction with the method, side effects, partner disagreement, and limited method options, lead many women to abandon contraception, resulting in higher unwanted pregnancies and a higher total fertility rate (10).

Injectable contraceptives, widely used for preventing pregnancy, particularly in sub-Saharan Africa (11–13), represent a major advance. Subcutaneous injectable contraception based on medroxyprogesterone acetate (DMPA-SC) (brand name Sayana® Press) represents a major advance, enabling self-administration and considerably widening access to contraception (14). It is an effective method, rivalling intrauterine devices, implants, and sterilisation. Moreover, the discreet nature of DMPA-SC is crucial, particularly for women living in rural communities who seek confidential family planning (15).

Self-care approaches, such as self-injection of contraceptives, have the potential to revolutionise healthcare systems and contribute to the achievement of universal public health. When integrated into primary healthcare, these self-care practices can significantly impact health and well-being (16). Self-injection of DMPA-SC or other injectable contraceptives could remove barriers and enhance contraception accessibility, facilitating its continued use, particularly for women who have difficulty gaining regular access to health facilities or where the availability of providers is limited (14, 17). Kim et al. found that self-administration of injectable contraceptives improves contraceptive compliance by eliminating the requirement of clinic visits for each injection (18).

Studies conducted in Burkina Faso, Niger, Senegal, and Uganda have demonstrated the favourable reception of DMPA-SC, paving the way for increased access to family planning for women who already use contraceptive methods, as well as new users and young women (16, 19–21). Economic analyses conducted in Senegal and Uganda suggest that self-injection can result in significant savings for women and health systems (16).

With a total fertility rate of 4.4 children per woman (22), Burkina Faso remains one of the countries with the highest fertility rate in the world. Despite an improvement in contraceptive prevalence, rising from 10% in 2010 (23) to 30.1% in 2021 (22), the most recent DHS data reveals that 13% of women still have unmet needs, and total demand for family planning remains low, with only 43% expressing a need for it (34% for birth spacing and 9% for contraception). Numerous barriers hinder the use of contraception, including poor access to services, women's marginalised status, high illiteracy rates among women, the patriarchal structure of society, and opposition from men (24, 25).

Increasing the supply of contraceptive methods through the introduction of DMPA-SC in 2014 and rapidly scaling up within public health facilities was a strategy implemented by the Ministry of Health to reduce barriers to women's access to family planning (26).

Since the introduction of self-administered injectable contraception, which offers the potential to address unmet family planning needs in developing countries, including Burkina Faso, numerous studies have been conducted. However, the main limitation of these studies lies in the use of samples that are not representative of the population of women of childbearing age who use contraceptive methods (27–28). Some studies recruited samples of women from health centres (29–31), which is not necessarily representative of the diversity of women nationwide. Others have used a cohort of women recruited during family planning campaigns organised in specific areas (32). Our study benefits from utilizing data sourced from the Demographic and Health Survey (DHS), ensuring the attainment of a nationally representative sample.

This study aims to understand the determinants that influence the use of DMPA-SC/Sayana Press among women who use modern contraceptive methods in Burkina Faso.

## 2 Methods

### 2.1 Study area

Burkina Faso is a landlocked country in the Sahel region, with a total area of approximately 272,969 km^2^. It shares borders with Mali to the northwest, Niger to the northeast, Benin to the southeast, and Côte d'Ivoire, Ghana, and Togo to the south. Administratively, Burkina Faso is divided into thirteen (13) regions and forty-five (33) provinces, which are, in turn, subdivided into 351 departments and 8,228 villages.

According to the results of the 5th General Census of Population and Housing (RGPH) in 2019, Burkina Faso had a total population of 20,505,155. This population breaks down into 9,900,847 men and 10,604,308 women, equivalent to a sex ratio of 107.1 women for every 100 men (34).

Women of childbearing age, i.e., those aged between 15 and 49, will represent 44.0% of the female population and 22.7% of the total population in 2019. Despite a downward trend in recent years, Burkina Faso has high fertility levels, comparable to other countries in sub-Saharan Africa.

### 2.2 Data source

This study uses secondary data obtained from the 2021 Burkina Faso Demographic and Health Survey (EDSBF). The survey employed a two-stage stratified sampling technique. In the first stage, clusters/enumeration areas (EAs) were selected from the national sampling frame, derived from the 2019 census. Details of the EDSBF methodology are described in detail in the EDSBF final report (22). The EDSBF sampling process is designed to ensure representativeness at the national level, considering the place of residence (urban, rural) and the regions of Burkina Faso.

The Demographic and Health Survey collects data on various health and social issues, including fertility, mortality, family planning, women's health, children's health, and men's health (35–37). This study focussed on a sample of women aged 15–49 who use modern contraceptive methods.

### 2.3 Study variables

#### 2.3.1 Dependent variable

The dependent variable was the use of DMPA-SC among women aged 15–49 who use modern contraceptive methods. Users of modern contraceptive methods are defined as women who use pills, intrauterine devices (IUDs), implants, injectable contraceptives, including DMPA/Sayana Press, male or female condoms, emergency contraception, female or male sterilisation, and other modern contraceptive methods. The dependent variable was coded as follows: 1 if the woman used DMPA-SC/Sayana Press, and 0 if she used any other modern contraceptive method.

#### 2.3.2 Independent variables

The explanatory variables used were selected in the light of the literature review (38–43) and include the woman's age, marital status, level of education, occupation, religion, parity, region and area of residence, media exposure, and her husband's level of education. In addition, variables such as discussion within the couple about family planning were considered in the analysis. Wealth quintiles and the question of distance to the health centre were also included in the analyses.

### 2.4 Data analysis

This study used both descriptive statistics and logistic regression models to analyse the data. The descriptive analysis used percentages to describe the study variables. Pearson's chi-square test was used to assess the associations between the explanatory variables and the study variable of interest. Bivariate logistic regression was used to examine the crude odds ratios of each explanatory variable to the dependent variable. The multivariate model was used to determine the net effect of each independent variable on the dependent variable. Significance levels were defined at *p* < 0.05, with corresponding confidence intervals. The results were presented in tabular form. Analysis was performed using Stata version 18 software.

### 2.5 Ethical considerations

This study is based on the analysis of secondary data without the use of any information enabling participants to be identified. The EDS survey was approved by ICF International and by a national ethics committee, and it received all the necessary authorisations to collect household data. All participants gave written informed consent before taking part in the survey. Although no additional ethical approval was required for this study, we obtained written permission from the DHS programme to use the data.

## 3 Results

In total, this study used a sample of 5112 women who use modern contraceptive methods. Table 1 presents the characteristics of the women according to the variables used in the study. DMPA-SC was used by 10.1% of women. The majority of the women included were aged between 15 and 29 (49.7%), followed by those aged between 30 and 39 (34.3%) and those aged between 40 and 49 (16.0%) and most women were Muslim (62.8%). The breakdown by marital status shows that a large proportion of women were married or living with a partner (82.2%), while 17.8% were not living with a partner.

**Table 1:** Socio-demographic characteristics of women using modern contraceptive methods.

Variables	*n*	%
Use DMPA-SC/Sayana		
Don't use DMPA-SC	4,618	90.3
Use DMPA-SC	494	9.7
Total	5,112	100
Age		
15–29	2,538	49.6
30–39	1,745	34.1
40–49	829	16.2
Total	5,112	100
Religion		
Muslim	3,184	62.3
Christian	1,696	33.2
Traditionnal	232	4.5
Total	5,112	100
Marital status		
Married/with partner	4,178	81.7
Not living with partner	934	18.3
Total	5,112	100
Type of residence		
Urban	2,063	40.36
Rural	3,049	59.64
Total	5,112	100
Administrative regions		
Boucle du Mouhoun	464	9.1
Cascades	312	6.1
Centre	612	12
Centre Est	451	8.8
Centre Nord	283	5.5
Centre Ouest	430	8.4
Centre Sud	378	7.4
Est	207	4
Hauts-Bassins	666	13
Nord	380	7.4
Plateau central	365	7.1
Sahel	178	3.5
Sud-Ouest	386	7.6
Total	5,112	100
Education attainment		
No education	2,841	55.6
Primary	768	15
Secondary or above	1,503	29.4
Total	5,112	100
Number of children		
No child	648	12.7
1–3	2,294	44.9
4–5	1,322	25.9
6 and above	848	16.6
Total	5,112	100
Discussion within the couple about FP and the number of children		
Yes	1,809	35.4
No	2,065	40.4
Not applicable	1,238	24.2
Total	5,112	100
Frequency on media (radio, tv, magazines)		
Not at all	1,178	23
Often or regularly	3,934	77
Total	5,112	100
Wealth index		
Poorest	623	12.2
Poorer	812	15.9
Middle	1,061	20.8
Richer	1,196	23.4
Richest	1,420	27.8
Total	5,112	100
Husband education level		
No education	2,798	54.7
Primary	613	12
Secondary/higher	767	15
Not applicable	934	18.3
Total	5,112	100
Women's occupation		
Inactives	1,546	30.2
Managers/employees	271	5.3
Female traders	1,166	22.8
Women farmers	1,602	31.3
Manual workers/housewives/domestic workers	527	10.3
Total	5,112	100
Problem of distance to get to a health centre		
Big problem	1,565	30.6
Not a big problem	3,547	69.4
Total	5,112	100

More than half of the women have no formal education (56.5%). Most women have between 1 and 3 children (45.0%), while 13.1% have no children yet. A significant proportion of women (40.2%) do not discuss family planning with their partner. The women have a variety of occupations, with a high proportion of farmers (30.6%) and shopkeepers (21.6%). For some women, the distance to a health centre is a significant problem (31.5%), which may have an impact on their ability to obtain contraceptive services.

Table 2 examines the associations between different explanatory variables and using DMPA-SC (Subcutaneous Depot of Medroxyprogesterone Acetate) in Burkina Faso. The results indicate significant associations with several demographic, socio-economic, and behavioural factors.

**Table 2:** Associations between explanatory variables and the use of DMPA-SC in Burkina Faso.

Variables	Use DMPA-SC/Sayana				
	Don't use	use	Total	*n*	*P*-value
Age					0.018
15–29	89.8	10.2	100	2,538	
30–39	89.8	10.2	100	1,745	
40–49	93.0	7.0	100	829	
Total	90.3	9.7	100	5,112	
Religion					0.647
Muslim	90.1	9.9	100	3,184	
Christian	90.6	9.4	100	1,696	
Traditionnal	91.8	8.2	100	232	
Total	90.3	9.7	100	5,112	
Marital status					0.001
Married/with partner	89.1	10.9	100	4,178	
Not living with partner	95.7	4.3	100	934	
Total	90.3	9.7	100	5,112	
Type of residence					0.001
Urban	92.29	7.71	100	2,063	
Rural	89.01	10.99	100	3,049	
Total	90.34	9.66	100	5,112	
Administrative regions					0.001
Boucle du Mouhoun	83.8	16.2	100	464	
Cascades	94.9	5.1	100	312	
Centre	89.1	10.9	100	612	
Centre Est	91.6	8.4	100	451	
Centre Nord	97.5	2.5	100	283	
Centre Ouest	90.5	9.5	100	430	
Centre Sud	93.7	6.3	100	378	
Est	91.3	8.7	100	207	
Hauts-Bassins	82.9	17.1	100	666	
Nord	90.0	10.0	100	380	
Plateau central	94.2	5.8	100	365	
Sahel	93.3	6.7	100	178	
Sud-Ouest	94.0	6.0	100	386	
Total	90.3	9.7	100	5,112	
Education attainment					0.046
No education	90.0	10.0	100	2,841	
Primary	88.7	11.3	100	768	
Secondary or above	91.7	8.3	100	1,503	
Total	90.3	9.7	100	5,112	
Number of children					0.001
No child	96.6	3.4	100	648	
1–3	89.1	10.9	100	2,294	
4–5	89.4	10.6	100	1,322	
6 and above	90.4	9.6	100	848	
Total	90.3	9.7	100	5,112	
Discussion within the couple about FP and the number of children					0.001
Yes	86.7	13.3	100	1,809	
No	91.0	9.0	100	2,065	
Not applicable	94.5	5.5	100	1,238	
Total	90.3	9.7	100	5,112	
Frequency on media (radio, tv, magazines)					0.421
Not at all	89.7	10.3	100	1,178	
Often or regularly	90.5	9.5	100	3,934	
Total	90.3	9.7	100	5,112	
Wealth index					0.001
Poorest	89.6	10.4	100	623	
Poorer	90	10	100	812	
Middle	89	11	100	1,061	
Richer	88.3	11.7	100	1,196	
Richest	93.6	6.4	100	1,420	
Total	90.3	9.7	100	5,112	
Husband education level					0.001
No education	89.2	10.8	100	2,798	
Primary	87.4	12.6	100	613	
Secondary/higher	90.4	9.6	100	767	
Not applicable	95.7	4.3	100	934	
Total	90.3	9.7	100	5,112	
Women's occupation					0.058
Inactive	89.7	10.3	100	1,546	
Managers/employees	91.9	8.1	100	271	
Female traders	90.8	9.2	100	1,166	
Women farmers	89.3	10.7	100	1,602	
Manual workers/housewives/domestic workers	93.4	6.6	100	527	
Total	90.3	9.7	100	5,112	
Problem of distance to get to a health centre					0.269
Big problem	89.6	10.4	100	1,565	
Not a big problem	90.6	9.4	100	3,547	
Total	90.3	9.7	100	5,112	

Table 2 examines the associations between different explanatory variables and the use of DMPA-SC (Subcutaneous Medroxyprogesterone Acetate Depot) in Burkina Faso. The results show significant associations with several demographic, socio-economic and behavioural factors.

The difference in DMPA-SC use between age groups was statistically significant (*p* = 0.018). The proportion of DMPA-SC users was higher among women aged 15–29 (10.2%) and 30–39 (10.2%) than among women aged 40–49 (7.0%). Marital status was associated with DMPA-SC use (*p* = 0.001). Women who were married or living with a partner (10.9%) had a significantly higher rate of use than those not living with a partner (4.3%).

There were also significant variations in the use of DMPA-SC according to administrative region (*p* = 0.001) and place of residence. The results show geographical disparities, with higher rates of use in some regions than in others. Women's level of education was another factor associated with the use of DMPA-SC (*p* = 0.046). The results show that women with secondary or higher education have a lower rate of use than those with no education or primary education (*p* = 0.046). The number of children already born was significantly associated with the use of DMPA-SC (*p* = 0.000). Women without children or with a higher number of children had a lower rate of DMPA-SC use. Women who discuss family planning within the couple are more likely to use DMPA-SC (*p* = 0.001). Wealth was significantly associated with DMPA-SC use (*p* = 0.001). The spouse's level of education was significantly associated with the use of DMPA-SC (*p* = 0.001).

Religion, place of residence, frequency of listening to the media, occupation of the woman, and the problem of distance to the health centre were not significantly associated with the use of DMPA.

Table 3 presents the results of the multivariate logistic regression analysis. The results of the logistic regression present the adjusted odds ratios (aOR) and 95% confidence intervals (95% CI) for different explanatory variables concerning the use of DMPA-SC. The age of the woman was significantly associated with the use of DMPA-SC. Women aged between 15 and 29 were twice as likely to use DMPA-SC as those between 40 and 49 (aOR = 2.12, *p* = 0.000). Women aged 30 to 39 were also more likely to use DMPA-SC than the reference group (aOR = 1.51, *p* = 0.02).

**Table 3:** Results of multivariate analysis of DMPA-SC use and explanatory variables among women using modern contraceptive methods in Burkina Faso.

Variables	Crude odds ratios (OR)		Adjusted odds ratios (aOR)	
	OR [95% CI]	*P*-value	aOR [95% CI]	*P*-value
Age				
15–29	1.50 [1.12, 2.02]	0,010	2.12 [1.42, 3.16]	0,001
30–39	1.51 [1.11, 2.06]	0,010	1.51 [1.08, 2.11]	0,020
40–49	1		1	
Religion				
Muslim	1		1	
Christian	0.95 [0.78, 1.16]	0,610	1.00 [0.80, 1.26]	0,970
Traditionnal	0.81 [0.50, 1.32]	0,400	0.93 [0.54, 1.60]	0,790
Marital status				
Married/with partner	2.72 [1.96, 3.80]	0,001	1.93 [1.22, 3.05]	0,010
Not living with partner	1		1	
Type of residence				
Urban	1		1	
Rural	1.48 [1.21, 1.80]	0,001	1.12 [0.85, 1.49]	0,420
Administrative regions				
Boucle du Mouhoun	7.60 [3.45, 16.75]	0,001	8.10 [3.64, 18.02]	0,001
Cascades	2.13 [0.86, 5.26]	0,100	2.82 [1.13, 7.04]	0,030
Centre	4.85 [2.20, 10.70]	0,001	7.78 [3.45, 17.53]	0,001
Centre Est	3.63 [1.60, 8.24]	0,001	3.56 [1.55, 8.14]	0,001
Centre Nord	1		1	
Centre Ouest	4.16 [1.84, 9.40]	0,001	4.47 [1.96, 10.20]	0,001
Centre Sud	2.67 [1.14, 6.29]	0,020	2.99 [1.26, 7.12]	0,010
Est	3.76 [1.54, 9.17]	0,001	3.17 [1.28, 7.87]	0,010
Hauts-Bassins	8.14 [3.74, 17.71]	0,001	10.49 [4.77, 23.06]	0,001
Nord	4.38 [1.93, 9.96]	0,001	4.62 [2.01, 10.59]	0,001
Plateau central	2.41 [1.01, 5.74]	0,050	2.46 [1.02, 5.91]	0,040
Sahel	2.85 [1.10, 7.38]	0,030	3.07 [1.16, 8.11]	0,020
Sud-Ouest	2.50 [1.06, 5.91]	0,040	2.60 [1.07, 6.34]	0,030
Education attainment				
No Education	1		1	
Primary	1.15 [0.90, 1.49]	0,270	1.20 [0.91, 1.58]	0,190
Secondary or above	0.81 [0.65, 1.01]	0,070	1.26 [0.94, 1.68]	0,120
Number of children				
No child	1		1	
1–3	3.50 [2.24, 5.45]	0,001	1.97 [1.09, 3.54]	0,020
4–5	3.37 [2.13, 5.34]	0,001	2.37 [1.23, 4.56]	0,010
6 children & above	3.00 [1.85, 4.87]	0,001	2.44 [1.21, 4.93]	0,010
Discussion within the couple about FP and the number of children				
Yes	1.56 [1.27, 1.91]	0,001	1.45 [1.17, 1.80]	0,001
No	1		1	
Not applicable	0.59 [0.44, 0.79]	0,001	0.71 [0.53, 0.96]	0,030
Frequency on media (radio, tv, magazines)				
Not at all	1		1	
Often or regularly	0.92 [0.74, 1.14]	0,420	1.10 [0.86, 1.39]	0,460
Wealth index				
Poorest	1		1	
Poorer	0.95 [0.67, 1.34]	0,780	0.93 [0.65, 1.34]	0,710
Middle	1.06 [0.77, 1.47]	0,700	0.97 [0.69, 1.37]	0,860
Richer	1.14 [0.83, 1.55]	0,420	1.01 [0.70, 1.47]	0,950
Richest	0.59 [0.42, 0.82]	0,001	0.47 [0.29, 0.76]	0,001
Women's occupation				
Inactive	1		1	
Managers/employees	0.77 [0.48, 1.23]	0,270	1.16 [0.69, 1.95]	0,580
Female traders	0.88 [0.68, 1.14]	0,340	1.03 [0.77, 1.37]	0,850
Women farmers	1.04 [0.83, 1.31]	0,720	0.94 [0.72, 1.21]	0,610
Manual workers/housewives/domestic workers	0.62 [0.42, 0.91]	0,010	0.69 [0.46, 1.03]	0,070
Problem of distance to get to a health centre				
Big problem	1		1	
Not a big problem	0.89 [0.73, 1.09]	0,270	0.95 [0.76, 1.18]	0,650
Constant			0.007 [0.001, 0.01]	0,001
*N*	5,112		**5,112**	

CI, confidence interval, OR, crude odds ratio, aOR, Adjusted odds Ratio, 1 = Reference.

Several regions showed a strong association with the use of DMPA-SC. For example, the Boucle du Mouhoun region had a high aOR of 8.10, which means that women in this region are significantly more likely to use DMPA-SC. Married women or those living with a partner [aOR = 1.93 (1.22, 3.05)] are more likely to use DMPA-SC. Women's level of education showed no significant association with the use of DMPA-SC.

Concerning the number of children the woman has, women with 1–3 children are approximately twice as likely to use DMPA-SC compared with those without children (aOR = 1.97, *p* = 0.02). Similarly, women with 4 to 5 children or 6 or more children were also significantly more likely to use DMPA-SC.

Women who discussed family planning within the couple were 1.45 times more likely to use DMPA-SC (aOR = 1.45, *p* = 0.001). Women for whom this discussion was not applicable were less likely to use it. The results show that women in the highest wealth group are significantly less likely to use DMPA-SC (aOR = 0.59, *p* = 0.001).

Women's occupations and problem of distance to get to a health centre showed no significant association with the use of DMPA-SC among women.

## 4 Discussion

This study aimed to understand the determinants influencing the use of DMPA-SC/Sayana Press among women who use modern contraceptive methods in Burkina Faso. The results show several significant associations between different explanatory variables and DMPA-SC use. These results provide crucial information to guide public health policies and awareness programmes aimed at improving access to this contraceptive method and promoting family planning in the country.

The studies showed that DMPA-SC users were more numerous among women aged 15–29 and those aged 30–39 than among women aged 40 and over. This result appears to be consistent with findings elsewhere. In a study in Uganda, Corneliess et al. found that, compared with adult women, adolescents were more likely to use a modern contraceptive method for the first time when they opted for self-injection (44). Also, in Uganda and the DRC, results showed that young women (15–19 years) were more likely to use DMPA-SC compared to those aged 45–49 years, which could result from DMPA-SC programmes targeting younger women in these contexts (33). Also, many current DMPA-SC users are first-time contraceptive users, suggesting that DMPA-SC is reaching new populations and potentially increasing the overall prevalence of modern contraceptives in some settings (33, 45). In addition, the fact that self-infection allows great discretion and fewer visits to health centres makes DMPA-SC an attractive method for young women and married and unmarried teenagers, particularly those in need of secret contraception (46). In addition, community distribution of DMPA-SC appears to be an effective service delivery model for hard-to-reach populations, those most at risk of unmet need for FP, and key populations such as young people and unmarried users (47) and aids in decreasing the discontinuation and abandonment of contraception usage (19, 48, 49).

Our results also showed that marital status is significantly associated with the use of DMPA-SC. Married women were more likely to use the Sayana press than women who were not in a union. This result is also consistent with previous research. In a study conducted in Burkina Faso, the Democratic Republic of Congo, and Uganda, the results showed that never-married women were more likely to use male condoms than DMPA-SC, which is consistent with research conducted elsewhere showing that never-married women were less likely to use DMPA-SC than all other modern methods combined. This may be because condoms are not generally used in marriage in sub-Saharan Africa (33).

The region of residence and the number of children per woman also play a role in the use of the DMPA-SC. Use of the DMPA-SC varies considerably from one region to another, with women living in the Hauts-Bassins, Boucle du Mouhoun, Centre, Centre West, and North being more likely to use the DMPA-SC than those living in other regions of the country. This variation between regions could be linked to the strategy for moving to the DMPA-SC in the health districts at the national level. Indeed, prior to the scale-up, the DMPA-SC was only available in four pilot health districts in Burkina Faso before being gradually extended to all health districts nationwide (26). Furthermore, Anglewicz et al., in their study, found that stocks of DMPA-SC varied according to context, with more regular stocks in private establishments in the DRC and public establishments in Burkina Faso and Nigeria (45). Also, persistent stock-outs and limited method options can disrupt contraceptive use and increase discontinuation (45).

This study emphasised the significance of parity (number of children per woman) in the use of DMPA-SC. Women with children were more likely to choose DMPA-SC than those without children. This result is consistent with the findings of Anglewicz et al. in both Uganda and the DRC, who found that women with two or more children (compared with no children or a single birth) were more likely to choose implants over DMPA-SC in both Uganda and the DRC (33).

The main strength of this study lies in its use of nationally representative data. However, several limitations were also identified. The cross-sectional nature of the study design prevents us from establishing a causal link between variables. In addition, the use of secondary data limited the integration of other determinants, such as quality of care, including advice on side effects, accessibility of services, and cultural factors, which were not considered in the models.

The results of the study serve as a starting point for further debate. In light of the results of this study, there are many crucial policy implications for improving access to and use of modern contraceptive methods in Burkina Faso. Policies should aim to increase education and awareness of contraceptive methods, with a focus on demographic groups least likely to use DMPA-SC, such as women aged 40–49. Specific information campaigns need to be developed for these groups. Also, the geographical disparities observed in the use of DMPA-SC require interventions to ensure equitable access to family planning services in all regions of the country. This could involve expanding services in regions where their use is less widespread.

## 5 Conclusion

Research into the use of DMPA-SC among women who use modern contraceptive methods in Burkina Faso has produced significant and informative results. The study, based on a representative sample of 5,112 women, revealed striking associations between several socio-demographic and behavioural variables and the use of this contraceptive method. The results of the study highlight significant differences in the use of DMPA-SC according to age, marital status, region of residence, level of education, number of children, and contraceptive discussion within the couple. Younger women are more likely to use DMPA-SC, as are those who are married or living with a partner. Geographical disparities and differences in the number of children a woman has are also significant factors in contraceptive use.

These results underline the need to adapt interventions to take account of socio-demographic, regional, and cultural differences to ensure an adapted and equitable service offer for the entire female population while addressing limitations and deepening understanding of the underlying determinants of DMPA-SC use. It is crucial to expand the distribution and enhance the accessibility of DMPA-SC to guarantee equal access to this family planning method in all regions. Additionally, we suggest intensifying awareness campaigns regarding this method, which would facilitate outreach to and enable marginalised women (rural, poor economic and social conditions) and young women to conveniently obtain DMPA-SC.

## Data Availability

Publicly available datasets were analyzed in this study. This data can be found here: http://dhsprogram.com/data/available-datasets.cfm.
